# Amine-Functionalized Gellan Gum-Based Hydrogel Loaded with Adipose Stem Cell-Derived Small Extracellular Vesicles: An In Vitro Proof of Concept for Enhancing Diabetic Foot Ulcer Healing

**DOI:** 10.3390/gels11020119

**Published:** 2025-02-06

**Authors:** Laura Tomasello, Mattia Biondo, Giuseppina Biscari, Luigi Di Rosa, Fabio Salvatore Palumbo, Calogero Fiorica, Giovanna Pitarresi, Sonya Vasto, Giuseppe Pizzolanti, Giorgio Arnaldi

**Affiliations:** 1 Laboratory of Endocrinology and Regenerative Medicine “Aldo Galluzzo”, Department of Health Promotion, Mother and Child Care, Internal Medicine and Medical Specialties (Promise), University of Palermo, 90127 Palermo, Italy; giuseppe.pizzolanti@unipa.it (G.P.); giorgio.arnaldi@unipa.it (G.A.); 2 Department of Biological, Chemical and Pharmaceutical Sciences and Technologies, University of Palermo, 90128 Palermo, Italy; mattia.biondo@unipa.it (M.B.); giuseppina.biscari@unipa.it (G.B.); luigi.dirosa@unipa.it (L.D.R.); fabiosalvatore.palumbo@unipa.it (F.S.P.); giovanna.pitarresi@unipa.it (G.P.); sonya.vasto@unipa.it (S.V.); 3 Advanced Technologies Network (ATeN) Center, University of Palermo, 90128 Palermo, Italy

**Keywords:** regenerative medicine, bioactive gels, diabetic foot ulcer, extracellular vesicles, adipose mesenchymal stem cells, biocompatible materials, wound healing, hydrogel

## Abstract

Diabetic foot ulcers (DFUs) are chronic wounds and a common complication of diabetes. A promising strategy in the treatment of DFUs involves the use of stem cell derivatives, such as small extracellular vesicles (sEVs), which can enhance cell proliferation and reduce inflammation while avoiding immunogenic responses. In this study, we evaluated the ability of adipose mesenchymal stem cell- (ASC)-derived sEVs to enhance the proliferation of human fibroblasts, which play a crucial role in wound regenerative processes. To mimic the inflammatory environment of DFUs, fibroblasts were cultured into the gellan gum (GG) modified with ethylenediamine (EDA) hydrogel scaffolds loaded with ASC-derived sEVs, under pro-inflammatory cytokines. Our comparative analysis demonstrated that sEVs loaded in GG-EDA hydrogel improved fibroblast viability in pro-inflamed conditions while retaining the anti-inflammatory and immunomodulatory properties of their cells of origin. By modulating the gene expression profile of fibroblasts to promote cell proliferation, wound healing and re-epithelialization, our system presents a promising therapeutic strategy for DFU healing.

## 1. Introduction

Diabetic foot ulcers (DFUs) are one of the most common complications of diabetes and consist of chronic ulcers involving different tissues, from the epidermis to the muscles and bones. The social and economic burden of these wounds establishes them as a critical public health concern, as they contribute to disability and generate significant healthcare costs [1]. Moreover, their healing is quite difficult and slow because of the neuroischemic alterations of diabetic patients that lead to a condition of chronic inflammation, reduced angiogenesis and higher odds of severe infections, resulting in an impaired wound healing process [2], making DFU treatment complex and not always successful. Nowadays, the management of DFUs relies on various strategies, including early and targeted antibiotic treatment for infected wounds. Additionally, wound care approaches, such as debridement, off-loading methods, hyperbaric oxygen therapy, negative pressure therapy and the use of gauzes, foams and specialized dressings, play a crucial role [3].

Each strategy has its own advantages and limitations and must be tailored to factors such as the severity of the lesion (e.g., depth, infection and exudation), patient characteristics and the risk of recurrence [3,4].

In the composite events of wound healing, fibroblasts are recognized as crucial in DFU treatment, for their functional role in migration, neovascularization and extracellular matrix deposition.

Dermal fibroblasts typically remain in a quiescent state under normal physiological conditions. However, they become activated during processes like tissue injury, inflammation or wound healing. Mechanical stress and signals from pro-inflammatory cytokines and factors released upon injury initiate a cascade of events that drive fibroblasts to undergo a phenotypic switch, producing an extracellular matrix (ECM), driving tissue repair and modulating immune response [5].

In DFUs, fibroblasts are dysfunctional because of an altered expression of genes involved in proliferation, migration and terminal activation, i.e., phosphatase tensin homolog (PTEN), profilin-1 (PFN-1), vascular epidermal growth factor (VEGF), metalloproteinases (MMP)-9 and Metallopeptidase Inhibitor (TIMP)-1, TGF (tumor necrosis factor)-β and IL (interleukin)-10 [6,7,8].

A downmodulated expression of PTEN, a gene involved in cellular proliferation and migration, was found in several diabetic complications, including nephropathy and DFUs [5,6]. Its inhibition results in an impairment of ECM composition with a reduction of collagen-1 amount and an unbalanced MMP-9/TIMP-1 ratio, leading to scarring [9,10].

PFN-1, a widely expressed actin-binding protein, is critical for cytoskeletal regulation and influences both physiological and pathological processes [11,12]. Evidence has shown significantly elevated pfn-1 levels in the serum and complication sites of diabetic patients, implying that reducing its levels may enhance tissue repair [13,14].

VEGF produced by fibroblasts induces angiogenesis, acting as a mitogen for endothelial cells, thus mediating wound healing. [15]. Moreover, activated fibroblasts can secrete immunomodulatory molecules such as TGF-β and IL-10, which suppress immune responses and support tissue homeostasis [16,17].

Studying the role of fibroblasts in diabetic foot ulcers (DFUs) and chronic wounds could advance the development of more effective strategies for wound healing. One promising approach involves the use of human multipotent stem cells and their derivatives. Among them, the extracellular vesicles (EVs), which have the potential to modulate fibroblast activity, reduce inflammation, enhance vascularization and promote tissue healing through their immunomodulatory properties [18,19,20]. Both stem cells and their EVs can stimulate tissue regeneration by using a biocompatible scaffold with a three-dimensional structure to support the healing process. Stem cells contribute to the formation of new tissue by colonizing the scaffold and through their ability to differentiate and proliferate. EVs, rich in bioactive molecules, stimulate the regenerative response of surrounding cells. This approach could create an optimal microenvironment that promotes the effective regeneration of damaged tissue [21].

Hydrogel scaffolds used to support cell proliferation must have properties that promote cell adhesion, growth and differentiation. Among them, the most promising materials are polysaccharide-based hydrogels and sponges. Hydrogels are three-dimensional polymer networks that can retain large amounts of water due to their highly cross-linked structure. This particular property makes them similar to the extracellular matrix (ECM) and ensures that cell proliferation is facilitated.

Specifically, sponges are dehydrated hydrogels that have the advantage of potentially absorbing much more exudate than hydrogels in their swollen form. The porous structure of these biomaterials also promotes cell penetration and nutrient flow, creating excellent conditions for cell proliferation [22,23]. This type of scaffold can be developed using various hydrogels and tailored to match the specific characteristics of both the wound and the patient. Additionally, it can be loaded with different bioactive substances, making it a promising example of personalized medicine for wound healing. [24,25,26,27]. In this study, we developed sponges made of Gellan Gum (GG), an anionic exopolysaccharide derived from the fermentation of *Sphingomonas elodea*, widely recognized as advantageous due to its exceptional biocompatibility and biodegradability, as well as its low cost [28,29]. In fact, GG is inert towards cells, as it lacks specific functional groups that allow for direct biological interactions. Although this aspect brings advantages such as biocompatibility, it limits its ability to promote cell adhesion. To overcome this limitation, chemical modifications can be made to the structure of GG, functionalizing it with groups capable of interacting with cells. Recently, a novel low molecular weight derivative of GG was developed by functionalizing polysaccharide with ethylenediamine (EDA). By exploiting the functionalized polymer, hydrogels with improved hydrolytic stability and higher elasticity have been obtained following the interaction of the amino groups with the glucuronic acid groups of the repeating unit of the tetrasaccharide [30,31].

Focusing on fibroblasts’ involvement in wound healing, our study aims to propose an in vitro model evaluating the immunomodulating and healing properties of EVs from adipose mesenchymal stem cells (ASCs) included in the GG-EDA scaffold for DFU treatment.

## 2. Results and Discussion

### 2.1. Small Extracellular Vesicles from Adipose Mesenchymal Stem Cells: Isolation and Characterization

One of the limitations of the use of GG-based wound dressing for chronic wounds is that they do not show antioxidant, anti-inflammatory or anti-bacterial properties. For this reason, we focused on the possibility of loading the hydrogel with small extracellular vesicles (sEVs)—nanoscale vesicles that can be abundantly isolated from mesenchymal stem cells, including adipose-derived mesenchymal stem cells (ASCs) and that may retain the beneficial properties of their cells of origin. ASCs were obtained from donors through a minimally invasive surgical procedure, making them suitable for autologous application. Moreover, their released sEVs offer an advantage over allogeneic use, as they are expected to provoke a reduced immunogenic response [32].

**Figure 1:**
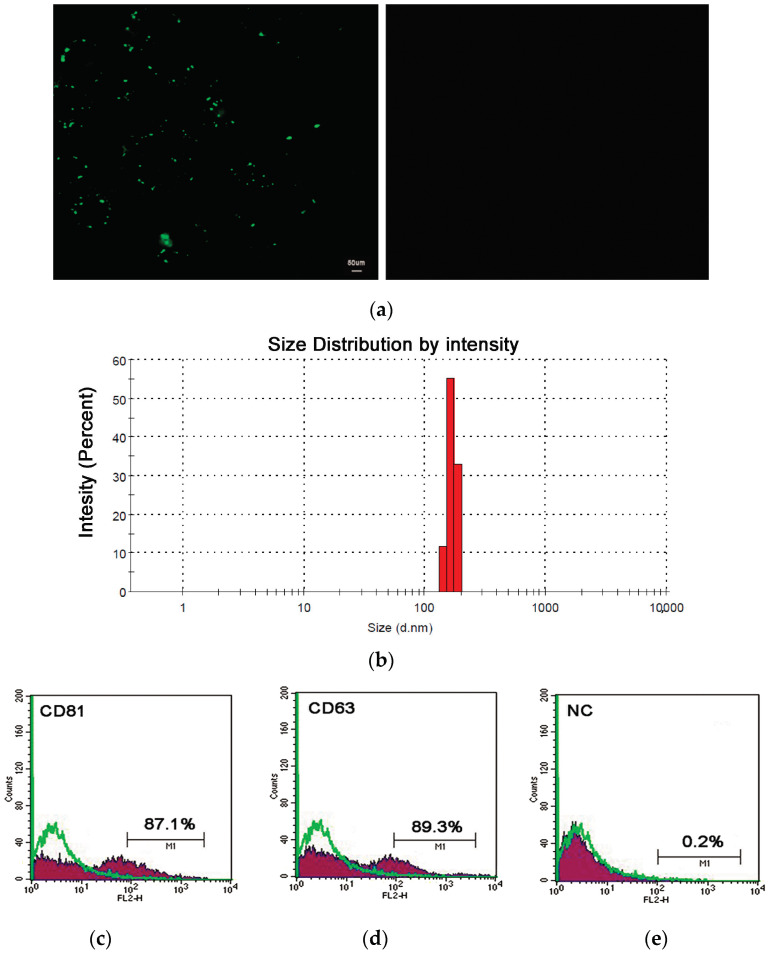
Characterization of small extracellular vesicles from adipose mesenchymal stem cells: (**a**) a representative immunofluorescent image of sEVs stained with CFSE (green fluorescence on left panel); (**b**) dynamic light scattering analysis: a representative intensity distribution graph of ASC-derived sEVs: (**c**–**e**) flow cytometry for sEV-specific markers: the histograms are representative of CD81 (**c**) CD63 (**d**) and positive cells. Cytometry control beads (**e**) and negative control, NC.

Using tangential flow filtration (TFF), the ASC-conditioned medium (CM) was ultrafiltered and concentrated up to 40 times. First, the concentrated suspension was stained with Carboxyfluorescein succinimidyl ester (CFSE) and observed under an immunofluorescent microscope. The immunofluorescence analysis revealed the presence of green-fluorescent nanoparticles with a likely diameter of less than 200 nm (Figure 1a).

To obtain a detailed size characterization of the isolated nanoparticles, a dynamic light scattering (DLS) analysis was conducted. The results indicated that the filtration device successfully isolated nanoparticles with a mean intensity distribution of 164.2 ± 24.1 nm (Figure 1b), which are classified as sEVs according to the guidelines of the International Society for Extracellular Vesicles (ISEV) [33]. Finally, the flow cytometric assay for CD81 and CD63, established surface markers for extracellular vesicles, confirmed that the ASC-derived nanoparticles were small extracellular vesicles (sEVs). The results showed that 78.95% ± 8.15% and 82.7% ± 6.6% of the gated events were positive for CD81 and CD63, respectively (Figure 1c,d).

### 2.2. Production and Characterization of GG-EDA Sponge

Gellan gum is a high molecular weight exopolysaccharide produced by microbial fermentation of *Sphingomonas elodea*: it has acquired a relevant role in biomedical applications due to its biocompatibility, biodegradability and non-toxicity.

Additionally, these properties can be improved with different functionalization strategies—such as the amine functionalization reported in this study—enhancing cell adhesion and proliferation to produce scaffolds suitable for regenerative medicine purposes. In our previous study, we investigated the effect of the presence of inter- and intra-polyelectrolyte interactions on the mechanical properties and physiological stability of GG-EDA hydrogel compared to GG. We previously evaluated GG-EDA hydrogel scaffolds, produced from a GG with a different molecular weight, and the results showed that it was able to ensure the viability of stem cells and promote osteogenic differentiation. The ability of the GG-based construct to balance fluid absorption while maintaining its integrity could make it ideal for applications in diabetic ulcer wounds, as it would be able to absorb exudate and at the same time help prevent tissue maceration [34].

**Figure 2:**
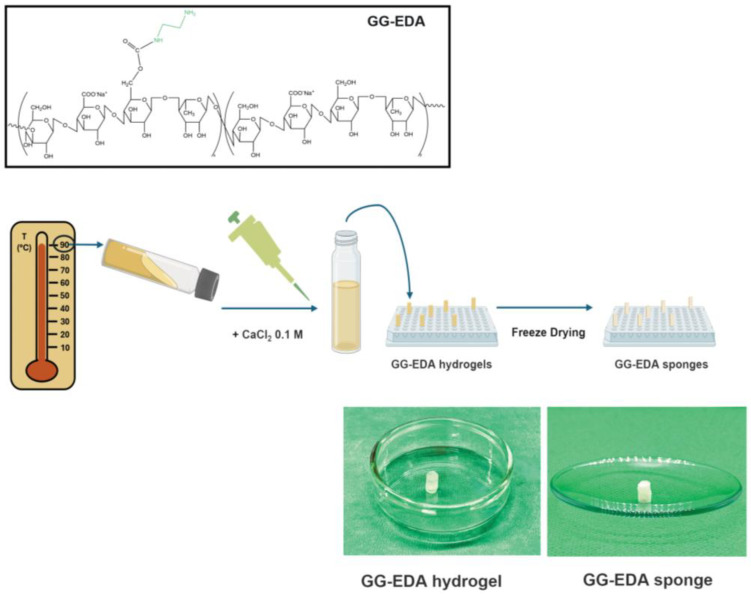
Schematic representation of production of GG-EDA sponges.

Here, the ionotropic cross-linking of the low molecular weight aqueous dispersion of GG-EDA was performed by injecting it into a 0.1 M CaCl_2_ solution. In this study, instead, the hydrogel was prepared by first dispersing the polymer at 6% in MilliQ water at 90 °C and directly injecting a 0.1 M CaCl_2_ solution. As shown in Figure 2, after ionotropic cross-linking, the hydrogel was freeze-dried, washed to remove excess calcium salt and freeze-dried again to obtain the GG-EDA sponge.

**Figure 3:**
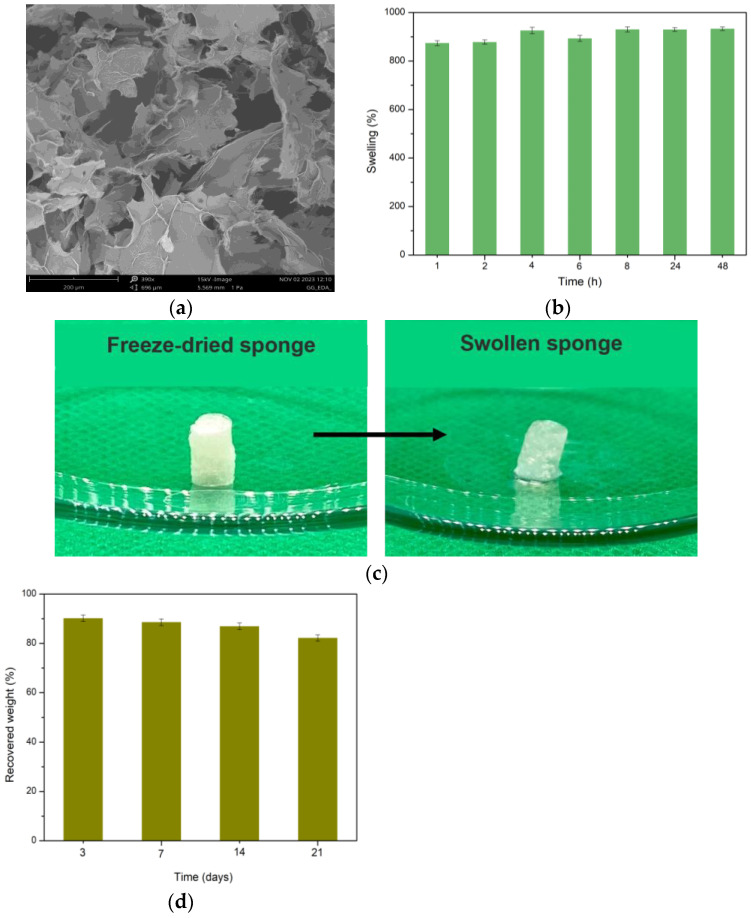
EM analysis (**a**), swelling % (**b**), photographs of dry and swollen samples (**c**), and hydrolytic degradation (**d**) of GG-EDA sponge. All data are shown as a mean value ± SD (n = 3).

Figure 3a shows the porous structure of the GG-EDA sponge, which is correlated to its adsorption capacity, which was evaluated through a swelling study (Figure 3b). The equilibrium swelling of the sponge was reached after about 8 h, when it reached a maximum swelling of more than 900%. Furthermore, although the scaffold absorbed a whole amount of medium, it managed to maintain its shape (Figure 3c). Regarding its hydrolytic stability, a degradation study was conducted (Figure 3d), which highlighted how stable the scaffold was in a physiological condition, losing less than 20% of its initial weight after 21 days.

### 2.3. GG-EDA Biocompatibility: HNDF Integration, Adhesion and Proliferation on GG-EDA Hydrogel Scaffolds

Biological safety and cytocompatibility are not the only critical factors when selecting a hydrogel for wound dressing: the extension of cell viability related to certain hydrogel is equally relevant. Indeed, the cells have to remain viable for several days within a scaffold placed in the wound in order to effectively exert their regenerative properties.

**Figure 4:**
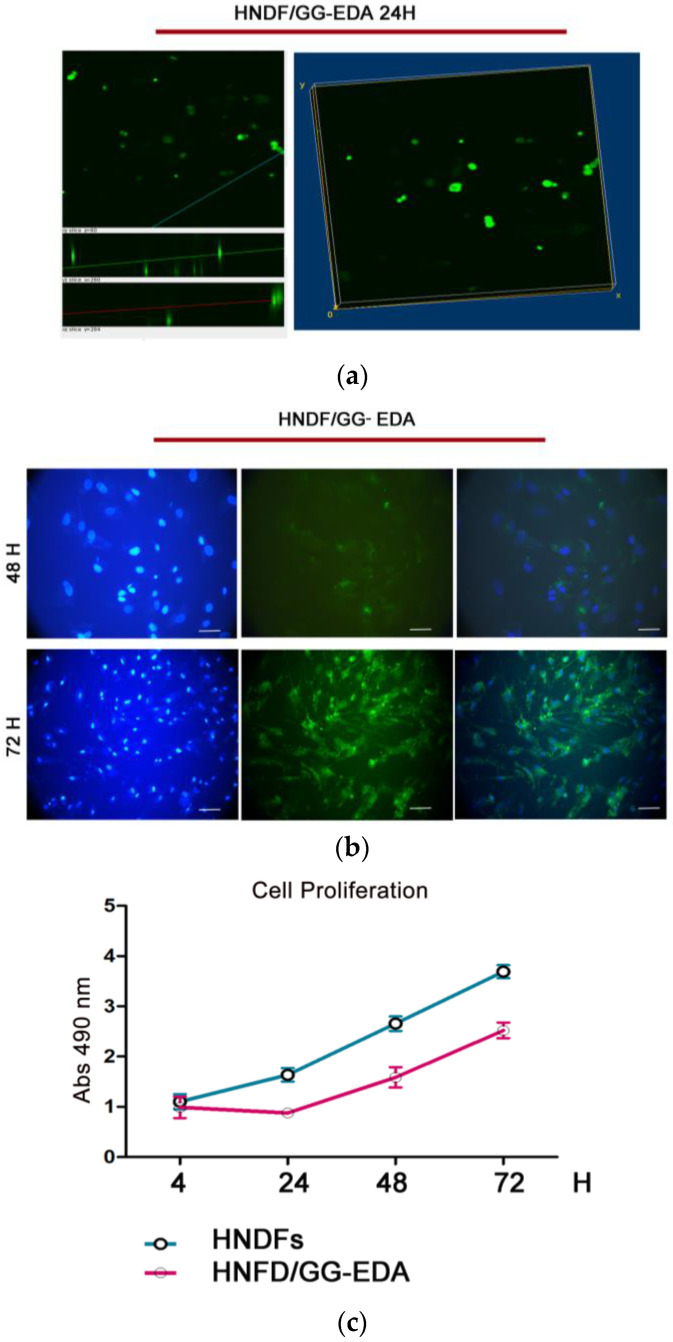
(**a**) Cell integration by confocal microscopy: a representative volume viewer by 3D reconstruction of HNDF at 24 h (40× magnification, scale bar: 20µm); (**b**) immunofluorescence microscopy exam of HNDF cells seeded in GG-EDA scaffold after 24 and 48 h (from left to right: DAPI staining, calcein AM staining and merged images); (**b**) cell viability: representative images of calcein AM staining of HNDF 48 and 72 h; (**c**) HNDF proliferation on GG-EDA compared to the control (HNDF) by MTS assay at 4, 24, 48 and 72 h. (Abs = absorbance at 490 nm). All experiments were performed in triplicate and repeated three times.

Given the critical role of fibroblasts in skin injury healing and regeneration [35], we conducted in vitro tests using a human fibroblast cell line (HNDF) to assess cell integration on GG-EDA hydrogel scaffolds. A confocal laser microscopy analysis was performed: at 24 h from the seeding procedure, data imaged by z-stacks showed rounded cells distributed into the scaffold (Figure 4a). Whereas at 48 and 72 h, immunofluorescence microscopy revealed cells with a more fibroblast shape, highlighting the capability of HNDF to spread into the hydrogel (Figure 4b). The direct biocompatibility test by MTS assay revealed a reduced growth rate of HNDF seeded on the GG-EDA scaffold when compared to HNDFs (0.115 vs. 0.168, respectively). However, despite the increment in doubling time (about 53 h vs. 41 h), the HNDF on the GG-EDA exhibited a proliferation trend comparable to the control group (Figure 4c). The initial inhibition of cell growth on the GGEDA is likely attributable to a physiological delay associated with the transition to suboptimal culture conditions compared to those optimal for adherent cells. This hypothesis is supported by the observed proliferative behavior in the subsequent days following the cells’ adaptation to the scaffold.

### 2.4. GG-EDA Loaded with ASC-Derived sEVs Enhances HNDF Proliferation by Exerting Immunomodulatory and Tissue Remodeling Effects in a Pro-Inflammatory Environment

Small extracellular vesicles (SEVs) represent a promising strategy for tissue regeneration; however, their short permanence in the damaged tissue limits their efficacy. To overcome this obstacle, in recent years many researchers have developed polymeric scaffolds enriched with SEVs, with the aim of improving their performance. A significant example is the study by Henriques-Antunes et al. [36]., who demonstrated that diabetic and non-diabetic wounds treated with a single dose of a photoactivatable injectable hyaluronic acid-based hydrogel containing sEVs showed a marked acceleration of closure compared to those treated with one or more doses of free sEVs. According to these findings, we decided to develop GG-EDA sponges that can be possibly enriched with EVs, promoting tissue healing. 

First, we conducted an enrichment with ASC-derived sEVs to investigate whether sEVs can positively affect the growth of cells in the GG-EDA scaffold. Tests were performed without and with pro-inflammatory cytokines mimicking the inflammatory microenvironment of DFUs. HNDFs were seeded on the GG-EDA hydrogel scaffolds enriched with 20 µg/mL of ASC-derived sEVs (GG-EDA + sEVs) and cultured up to 48 h under pro-inflammatory conditions, with a 20 ng/mL of TNF-α and 20 ng/mL of IL-1β.

**Figure 5:**
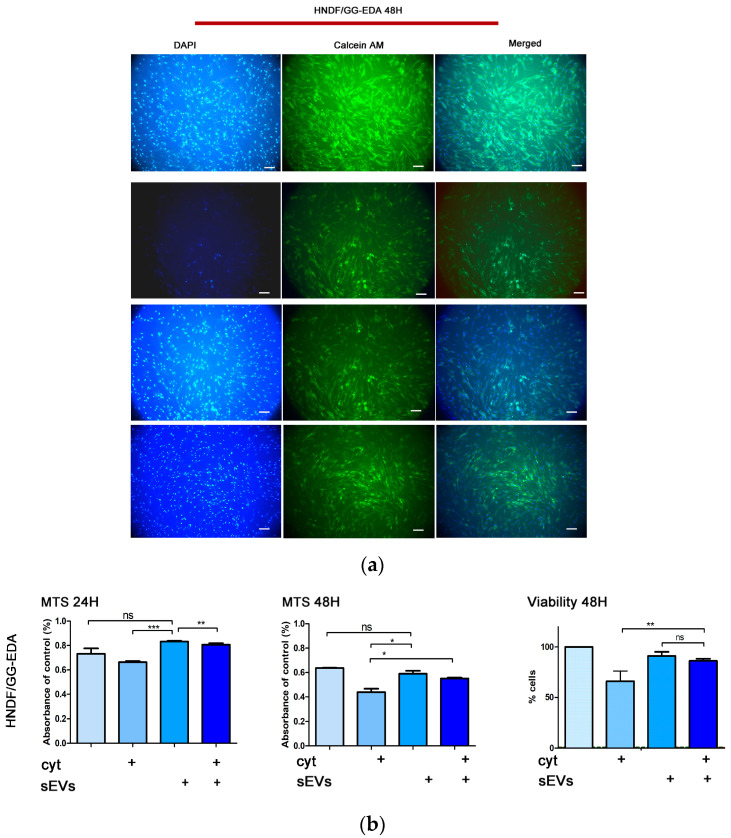
(**a**) Comparative immunofluorescence microscopy exam of pro-inflamed HNDF cells seeded on GG-EDA with or without sEVs scaffold at 48 h and HNDF cells (from left to right: DAPI staining, calcein AM staining and merged images) (20× magnification, scale bar: 20 µm); (**b**) cell viability: histograms representing MTS assay at 24 h, MTS assay at 48 h and overall rescue of viability (from left to right) of pro-inflamed HNDF cells seeded on GG-EDA scaffold with or without sEVs at 48 h. Data are expressed as mean ± standard deviation. (* *p* < 0.05, ** *p* < 0.001, *** *p* < 0.0001, *p* > 0.05 n.s.). All experiments were performed in triplicate and repeated three times.

GG-EDA enriched with ASC-derived sEVs treated with cytokines (GG-EDA + sEVs + cyt) were observed under an immunofluorescent microscope after 48 h of culture. Immunofluorescence calcein AM staining suggested an improvement in the cell density in HNDF/GG-EDA + sEVs + cyt compared to HNDF/GG-EDA + cyt (Figure 5a).

The MTS assay at 24 and 48 h confirmed a significant advantage of cell viability when pro-inflamed HNDF were cultured on GG-EDA + sEVs (*p* = 0.0018, and *p* = 0.022, Figure 5b). Analyzing the overall potential rescue effect of sEVs after 48 h, we found a significant (*p* = 0.0016) increment of cell viability of up to 84.21% ± 1.15% vs. 63.67 ± 1.453% in HNDF/GG-EDA + cyt + sEVs when compared to HNDF/GG-EDA + cyt (Figure 5b right panel).

Inflammation can hinder wound healing by altering the expression of genes involved in the regulation of the cell cycle, thereby impairing cellular turnover. It can also affect genes involved in tissue remodeling, obstructing the migration of cell types responsible for lesion closure, as well as genes associated with the inflammatory response itself.

**Figure 6:**
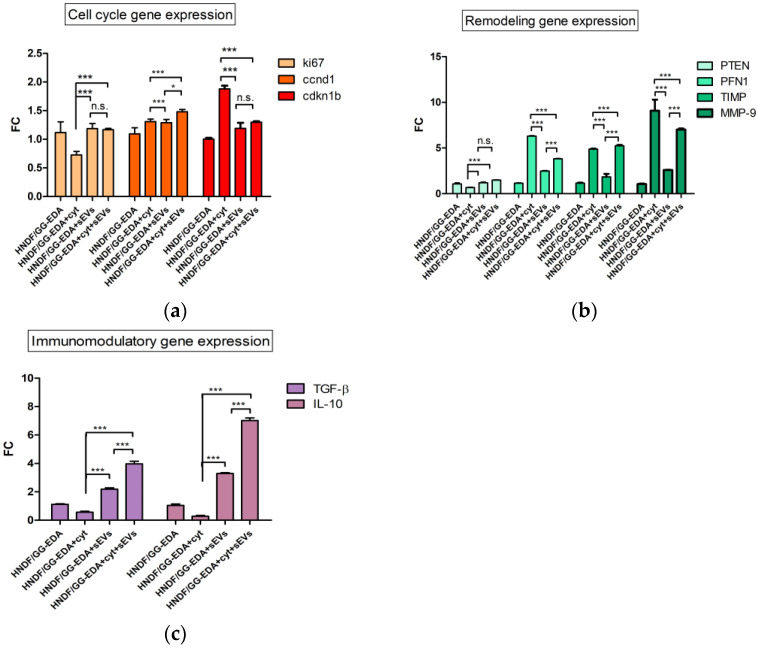
Comparative gene expression analysis of pro-inflamed HDNFs seeded on GG-EDA hydrogels with or without sEVs: (**a**) histogram representing gene involved in cell cycle regulation; (**b**) histogram representing gene involved in cytoskeleton remodeling; (**c**) and histogram representing gene involved in immunomodulatory response. Data are expressed as mean ± standard deviation. (* *p* < 0.05, *** *p* < 0.0001, *p* > 0.05 n.s.). All experiments were performed in triplicate and repeated three times.

Focusing on genes involved in cell proliferation (Ki67, ccndn1 and cdkn1b), cytoskeletal remodeling (PTEN, PFN-1, TIMP-1 and MMP-9), wound healing and immune homeostasis (TGF-β and IL-10), we performed a gene expression analysis. In detail, Ki67 and ccnd1 mRNA levels were found significantly reduced in the pro-inflamed HDNF seeded on the GG-EDA scaffold when compared to pro-inflamed HDNFs seeded on the GG-EDA + sEVs scaffold (1.167 ± 0.024 vs. 0.725, *p* < 0.0001 ± 0.024 and 1.48 ± 0.042 vs. 1.31 ± 0.042, *p* < 0.0001 respectively, Figure 6a), while an opposite trend was found in cdkn1b mRNA levels (1.29± 0.016 vs. 1.88 ± 0.057, *p* < 0.0001, Figure 6a). PTEN mRNA levels were found higher in the pro-inflamed HDNF seeded on the GG-EDA + sEVs scaffold when compared to the HDNF seeded on the GG-EDA scaffold in pro-inflammatory conditions (1.499 ± 0.016 vs. 0.675 ± 0.025, *p* < 0.0001, Figure 6b). On the contrary, a decrement was detected in PFN-1 mRNA levels in the pro-inflamed HDNFs seeded on the GG-EDA scaffold when compared to the pro-inflamed HDNFs seeded on the GG-EDA + sEVs scaffold (3.820 ± 0.030 vs. 6.310 ± 0.030, *p* < 0.0001, Figure 6b). Moreover, the TIMP-1/MMP-9 ratio was found twofold in the pro-inflamed HDNFs seeded on the GG-EDA + sEVs scaffold when compared to the pro-inflamed HDNFs seeded on the GG-EDA scaffold (0.0088 vs. 0.0044, *p* < 0.0001, Figure 6b). Finally, TGF-β and IL-10 mRNA levels were found significantly increased in pro-inflamed HDNFs seeded on the GG-EDA + sEVs scaffold when compared to the pro-inflamed HDNFs seeded on the GG-EDA scaffold (3.975 ± 0.177 vs. 0.565 ± 0.064, *p* < 0.0001 and 7.020 ± 0.184 vs. 0.28 ± 0.0570, *p* < 0.0001 respectively, Figure 6c).

Collectively, these results confirm that our construct effectively exerts a protective effect against inflammation, preserves cell viability and could enhance wound repair [33,37,38,39,40]. We observed a proliferative advantage in cell proliferation in the hydrogel scaffolds if the constructs are enriched with sEVs after treatment with inflammatory cytokines [33]. Although the regenerative and immunomodulant properties of sEVs are largely accepted, there is a lack of consensus about the specific mechanisms through which they exert these effects. As regards the transcription of genes related to immunomodulation, our data showed a higher expression of PTEN, acting as tumor suppressor but also as a keeper of immune homeostasis in HDNFs seeded on GG-EDA + cyt + sEVs [41]. Moreover, the downregulated mRNA levels of PTEN have been related to diabetic complications, explaining the outcome registered in HDNFs seeded on GG-EDA + cyt [41,42]. Additionally, while MMP-9 tends to increase in an inflammatory event [43], TIMP-1 has the role of limiting MMPs’ action, balancing the pro-inflammatory stimuli. We detected higher levels of MMP-9 in HDNFs seeded on GG-EDA + cyt, because of the uncontested action of the pro-inflammatory cytokines. Indeed, some authors stated that the imbalance between these factors can lead to pathological conditions [43,44]. This was in line with our findings that showed an improvement in the TIMP-1/MMP-9 ratio in pro-inflamed HDNFs seeded on GG-EDA+ sEVs. Moreover, there is a significant reduction of PFN-1 expression in HDNFs seeded on GG-EDA + cyt + sEVs than in HDNFs seeded on GG-EDA + cyt: this peculiar data could be related to its function of cytoskeleton remodeling, which is improved in fibroblasts under stress because of a pro-inflammatory event [44]. Finally, TGF-β—re-epithelialization promoter—and the IL-10—anti-inflammatory mediator—were found to have significantly increased in the pro-inflamed HDNFs seeded on the GG-EDA + sEVs compared to pro-inflamed HDNFs seeded on the GG-EDA. This finding suggests a potential role of the construct in enhancing injury repair [45,46].

## 3. Conclusions

Chronic wounds, such as DFUs, are a relevant health topic because of their slow healing and impaired regeneration. While therapies based on stem cells in situ applications to improve tissue regeneration are one of the promising strategies to treat chronic lesions, they could present some issues related to the eventual immunogenic response—especially if the cells do not derive from an autologous sampling—or to a potential malignant degeneration. Cell-free therapy involving only cell derivatives, such as the sEVs exerting protective, anti-inflammatory and immunomodulatory properties could limit eventual immunological responses after its administration.

Thus, our study aims to prove the feasibility of this kind of therapy after EV administration via GG hydrogels [47]. We focused on the possibility to use sEVs derived from adipose mesenchymal stem cells (ASCs) to enrich a Gellan Gum (GG)-based scaffold functionalized with ethylenediamine (GG-EDA), following the production of an ionotropically cross-linked and then freeze-dried hydrogel.

Our construct was able to enhance wound repair and promote cell proliferation in an inflammatory environment thanks to sEVs’ ability to modulate the gene expression of anti-inflammatory and immunomodulatory molecules [40,48].

A complete analysis of sEVs’ content and further experiments are necessary to better understand the mechanisms of EV-mediated wound healing and their relationship with the immune system. In fact, while we have directly evaluated the protective effect of sEVs in an inflammatory environment, a relevant limitation of this study consists of the need for a deep characterization of their content, which could offer new perspectives about their role. Additionally, a detailed in vivo study could be beneficial, not only to assess the real advantage of the GG-EDA hydrogel scaffolds loaded with sEVs, but to evaluate the real effects of sEVs on the immune system. In fact, while it is true that EV cell-free therapy could limit an immunogenic response, the relationship between EVs and immune cells is quite complex and sometimes could also include pro-inflammatory stimuli caused by the EV-mediated activation of innate immune cells [32,39].

## 4. Materials and Methods

### 4.1. Patient Selection

The eligibility criteria for participants were as follows: overweight patients (males and females) with no known allergies to drugs or foods, no consumption of drugs as chronic therapy or food supplements. Written consent about the use of the biological samples was given by each patient, in accordance with the Declaration of Helsinki. Patients’ features were reported in Table 1.

### 4.2. Adipose Tissue Collection

The adipose tissue (AT) was obtained after an elective surgical procedure of abdominoplasty from overweight patients. All volunteer patients had undergone a complete pre-operative assessment before undergoing surgery. Furthermore, they did not have cardiovascular or dysmetabolic pathologies. During the abdominoplasty, 10 mL of lipoaspirate was collected through a multiport microcannula with 3 mm holes, to obtain clusters of AT ready to be subsequently processed. AT was collected for all patients from the abdominal region immediately below the navel, at a depth of approximately 4 cm from the skin surface.

### 4.3. Cell Culture

#### 4.3.1. Adipose Mesenchymal Stem Cell Isolation and Cultivation Cell Procedures

Adipose mesenchymal stem cells (ASCs) were isolated from AT obtained after the surgical procedure. AT was first mechanically processed and cut into small pieces, removing small blood vessels and fibrous tissues. Then, it was placed in 50 mL polypropylene tube (Corning, Thermo Fisher Scientific Inc., Segrate (MI), Italy) and incubated at 5% CO_2_ and 37 °C for enzymatic digestion with collagenase type I (Gibco, Thermo Fisher Scientific Inc., Segrate (MI), Italy, as previously described [49]. After an incubation of 4 h, the isolated stromal vascular fraction (SVF) was placed in culture flasks and incubated with Dulbecco’s Modified Eagle Medium (DMEM/HAM’S F12, Euroclone S.p.A, Pero (MI), Italy) with stable L-glutamine supplemented with 10% of fetal bovine serum (FBS, Euroclone S.p.A, Pero (MI), Italy). In the following days, the culture medium was carefully changed every three days to remove the remaining free fat. In a few days, ASCs reached confluence, and subculturing was set up. Before they were used in the experiments, primary ASCs were characterized by the expression of the main mesenchymal stem cell-specific markers (Supplementary Figure S1). Next, ASCs were used at passage 2–4 (p2, p4) for all experiments.

#### 4.3.2. Primary Human Normal Dermal Fibroblast Cell Culture

Primary dermal, normal, human, neonatal (HNDFs) fibroblasts were purchased by ATCC (catalogue number PCS-201-010, LGC Standards S.r.l., Sesto San Giovanni, Italy). HDNFs were seeded in a T25 flask culture and kept in DMEM/HAM’S F12 (Euroclone S.p.A, Pero (MI), Italy) with stable L-glutamine supplemented with 10% FBS (Euroclone S.p.A, Pero (MI), Italy) and 10 ng/mL basic FGF (b-FGF, PeproTech, Thermo Fisher Scientific Inc., Segrate (MI), Italy). Culture and subculture were set up according to the ATCC handling procedure. Cells were used until the 5th culture passage (p5).

### 4.4. Small Extracellular Vesicles (sEVs)

#### 4.4.1. sEVs Isolation by Tangential Flow Filtration

To extract the small extracellular vesicles (sEVs) from cells, ASCs were expanded to passage 4 and cultured to achieve a confluence of >80%. Then, the culture medium was discarded, and the flasks were washed twice with PBS. The cells were placed in serum starvation conditions overnight. On the day after, 40 mL of conditioned medium was collected, and the sEVs were concentrated in 1 mL-aliquots via a tangential flow filtration (TFF) using a TFF-easy concentrator 5 (HansaBioMed, Tallinn, Estonia), capable of isolating vesicles until 200 nm in size.

#### 4.4.2. Dynamic Light Scattering (DLS)

Size distribution and quantification of sEVs: suspension of ASC-derived sEVs were acquired by a Zetasizer Nano ZSP2 instrument (Malvern, UK). Samples were diluted at a ratio of 1:100 in water, and size distribution and quantification of sEVs were detected by dynamic light scattering (DLS) as the intensity was measured on the Zetasizer v.7.11 software (Malvern Panalytical Srl, Lissone (MB), Italy). The measurement parameters were measurement type (size); material (polystyrene latex); material refractive index (RI, 1.59); absorption (0.01); dispersant (water); dispersant RI (1.33); temperature (25 °C); viscosity (0.8872 cP); duration (60 s); measurement position (3); cell description disposable micro cuvette (40 μL); and data processing size (distribution by intensity). The amount of sEVs was assessed via a Qubit Protein Assay Kit and Qubit fluorometer (by Invitrogen, Thermo Fisher Scientific Inc., Segrate (MI), Italy), according to manufacturer instructions.

#### 4.4.3. Flow Cytometry Analysis

The sEVs markers, CD63 and CD81, were determined using Exo-FACS according to the manufacturer’s instructions (HansaBioMed Life Sciences, Tallinn, Estonia). All reaction mixtures were then acquired using a FACS Calibur flow cytometer (Becton-Dickinson, Franklin Lakes, NJ, USA) and analyzed with the CellQuest Pro software v 4.01 (Becton-Dickinson, Franklin Lakes, NJ, USA).

#### 4.4.4. Immunofluorescence Analysis

sEVs were labelled with luminal binding dye 5-(and-6)-Carboxyfluorescein Diacetate Succinimidyl Ester (CFSE) according to manufacturer instructions (CellTrace CFSE Cell Proliferation Kit Thermo Fisher Scientific Inc., Segrate (MI), Italy). Briefly, a 5 mM of CFSE stock solution was prepared by adding 18 µL of dimethyl sulfoxide (DMSO, Sigma-Aldrich/Merck, Darmstadt, Germany) to a vial of CellTrace reagent. To stain sEVs with CFSE, 1 µL of CFSE stock was added to 300 µL of PBS prior to staining, and then 1 µL of sEVs from 100 µg/mL sEVs suspension was added and incubated for 2 h at 37 °C [50]. sEVs were observed under a Leica DMI300B inverted fluorescence microscope (Leica, Wetzlar, Germany), and the images were analyzed using Leica Application Suite X, LAS X (Leica, Wetzlar, Germany).

### 4.5. Gellan Gum Scaffold Preparation

Low molecular weight Gellan Gum (GG) was obtained from high molecular weight GG (Gelzan™ CM, Merck, Rome, Italy) under alkaline conditions for 8 h, as previously reported [30]. To facilitate its dispersion in organic solvents, tetrabutylammonium salt (GG-TBA) was prepared [30]. The synthesis of the Gellan gum-((2-aminoethyl)-carbamate) (GG-EDA) derivative was carried out with modifications to the previously described procedures [30]. The derivative used in this work had a functionalization degree in EDA moieties equal to 28 ± 4 mol% calculated by a 1H NMR analysis by comparing the area of the integrals at δ 2.6 ppm and the integral of the peak at δ 1.2 ppm representing the protons of the methyl group of the rhamnose of GG. The degree of molar functionalization in EDA was also confirmed by a 2,4,6-trinitrobenzene sulfonic acid (TNBS) assay. The GG-EDA was dispersed in Milli-Q water at a concentration of 6% *w*/*v* at 90 °C. Specifically, 60 mg of GG-EDA was dispersed in 1 mL of Milli-Q water. While the mixture was still hot, 100 μL of 0.1 M CaCl2 was added and vortexed for 60 s. The hydrogels obtained by the ionotropic cross-linking of the GG-EDA were cut (giving 10 hydrogels of about 100 uL each), frozen at −80 °C and freeze-dried to give GG-EDA sponges. The dried hydrogels were washed five times in an orbital shaker at 37 °C for 10 min each time with 2 mL of Milli-Q water to remove excess calcium salt. Finally, the samples were freeze-dried again.

### 4.6. SEM Analysis of GG-EDA Sponge

A scanning electron microscopy (SEM) image was acquired with a Phenom XL instrument, Alfatest (Thermo Fisher Scientific Inc., Segrate (MI), Italy). The image was obtained after the precursor hydrogel was frozen in liquid nitrogen, cut with a sharp blade and only then freeze-dried.

### 4.7. Swelling of GG-EDA Sponge

GG-EDA sponge (prepared from 100 μL hydrogel) was first weighed dry and then incubated in 2 mL DPBS pH 7.4 at 37 °C. After each time point (1, 2, 4, 6, 8, 24, 48 h) the swollen scaffold was weighed after removing the excess of buffer with paper. The swelling percentage (Sw%) was calculated as

(1) $$Sw\%=\left(\frac{Wsw-Wd}{Wd}\right)\times 100$$

where *Wsw* is the weight of the sponge after swelling, and *Wd* is the weight of the dry sponge. Each experiment was performed in triplicate and the results were expressed as mean value ± standard deviation.

### 4.8. Hydrolytic Degradation Study of GG-EDA Sponge

The hydrolytic degradation study was performed by weighing the freeze-dried sample and then incubating it in DPBS pH 7.4 at 37 °C. At scheduled time intervals (3, 7, 14 and 21 days), the sample was frozen, freeze-dried and weighed. The hydrolytic degradation of the sample was expressed as a percentage of the recovered weight (Wr%), calculated as

(2) $$Wr\%=\left(\frac{Wf}{Wi}\right)\times 100$$

where *Wf* is the weight of the degraded sample at each time point, and *Wi* is its initial weight. Each experiment was performed in triplicate and results were expressed as mean value ± standard deviation.

### 4.9. Sponge Sterilization

Following the production of the GG-EDA sponge by freeze-drying and prior to further studies in which it would be used as a scaffold for cells and EVs, sterilization was performed by UV irradiation at 254 nm for at least 1 h (30 min per side of sample) using a 125 W UV lamp.

### 4.10. Biocompatibility

#### 4.10.1. Cell Seeding

After reaching confluence, HNDFs were trypsinized, and 5 × 10^4^ cell/cm^2^ were seeded on the dry GG-EDA scaffold surface. After seeding, each scaffold was loaded with a final volume of 100 µL of the culture medium.

#### 4.10.2. Cell Viability Assay

The cell metabolic activity was evaluated using3-(4,5-dimethylthiazol-2-yl)-5-(3-carboxymethoxyphenyl)-2-(4-sulfo-phenyl)-2H-tetrazolium (MTS, Sigma-Aldrich, Merck Italy, Rome, Italy) after 4 h of incubation at 37 °C in 5% CO_2_. Absorbance was measured at 24, 48 and 72 h in a microplate reader (SpectroStarNano, BMGLABTECH, Ortenberg, Germany) at 490 nm. Measurements were analyzed by the Mars Software (BMG LABTECH, Offenburg, German) and represented as growth curves via GraphPad 5.0 Software, Inc., La Jolla, CA, USA

#### 4.10.3. Cell Visualization on Hydrogel Scaffolds

HNDFs were seeded at a density of 5 × 10^4^ cell/cm^2^, the cell integration capability was evaluated at 24 h by calcein AM (C-AM) staining according to the Thermo Fisher staining protocol. The fluorescent staining was acquired using a confocal microscope Olympus FV10i (Leica microsystems Srl, Buccinasco (MI), Italy) at 24 h. The acquired images were reconstructed with an Imagej2 Software (version 2.0.0-beta-7.1) by a volume viewer project, based on a z-stack measure. Cell adhesion was evaluated at 48 and 72 h by fluorescent microscope observation using a Leica DMI300 instrument (Leica microsystems Srl, Buccinasco (MI), Italy).

#### 4.10.4. Cell Viability on GG-EDA Scaffold

HNDFs were seededate a cell density of 5 × 10^4^ cell/cm^2^ on GG-EDA or on a 96-well cell culture plate, and cell viability was evaluated using 3-(4,5-dimethylthiazol-2-yl)-5-(3-carboxymethoxyphenyl)-2-(4-sulfo-phenyl)-2H-tetrazolium (MTS) and assessed with the MTS assay (CellTiter 96^®^ AQueous One Solution Cell Proliferation Assay, by Promega (Milan, Italy)at 4, 24, 48 h and 72 h in a microplate reader (SpectroStarNano, BMGLABTECH, Ortenberg, Germany) at 490 nm. The measures of the absorbances were analyzed by the Mars Software v.o. 1.10 (BMG LABTECH, Offenburg, German) and represented as a histogram on GraphPad Software 5.0, Inc., La Jolla, CA, USA.

### 4.11. GG-EDA Hydrogel Scaffold Enrichment

The amount of ASC-derived sEVs was established by assessing a two-step protocol by using an ExoQuick-TC precipitation solution (System Biosciences, Palo Alto, CA, USA) and a Qubit™ Assay (Thermo Fisher Scientific Inc., Segrate, MI, Italy) on a Qubit fluorometer instrument. Briefly, 2 mL of ExoQuick TC buffer was added to 10 mL of a condition medium and was mixed and placed at 4 °C overnight. On the next day, after a centrifugation at 1500 rpm, 200 µL of Exo Binding buffer was added to the pellet. Finally, 10 µL of the sample was quantified by a Qubit™ Assay according to the manufacturer’s instructions (Thermo Fisher Scientific Inc., Segrate (MI), Italy).

Each GG-EDA scaffold was enriched with a suspension of 20 µg/mL of ASC-derived sEVs.

### 4.12. Functional Studies

#### 4.12.1. Pro-Inflammatory Cytokines Conditions

20 ng/mL of tumor necrosis factor (TNF-α) and 20 ng/mL of interleukin 1 beta (IL-1β) were prepared. Both cytokines were provided by Peprotech (Thermo Fisher Scientific Inc., Segrate (MI), Italy).

Cell viability on the GG-EDA scaffold was enriched with small extracellular vesicles (GG-EDA + sEVs)

HNDFs were seeded at 5 × 104 cell/cm^2^ on the GG-EDA and the GG-EDA + sEV hydrogel scaffolds under pro-inflammatory conditions. Cell viability was calculated at 24 and 48 h via an MTS assay (CellTiter 96^®^ AQueous One Solution Cell Proliferation Assay, by Promega, Milan, Italy). The potential rescue effect of EVs was calculated as the difference in viability percentage compared to the cytokine-treated HNDFs cultured on the GG-EDA (without sEVs) at 48 h.

#### 4.12.2. Gene Expression Analysis

The total RNA from HNDFs was extracted and purified using the RNeasy Micro Kit (Qiagen, Milano, Italy), according to the manufacturer’s protocol. Quantitative and qualitative analyses (260/280 nm of absorbance) were performed on a Nano Drop 2000 spectrophotometer (Thermo Fisher Scientific Inc., Segrate (MI, Italy). An amount of 2 µg of total RNA was reverse transcribed in a final volume of 20 µL with Oligo dT primers (Promega Italia s.r.l., Milano, Italy) and ImProm-II™ Reverse Transcription System (Promega Corporation, Madison, WI, USA). The Quantitect SYBR Green PCR Kit (Qiagen, Milano, Italy) was used for real time quantitative analysis by RotorGene Q Instrument (Qiagen, Milano, Italy). Briefly, amplification conditions were the following: 95° C for 3 min, 40 cycles at 95 °C for 20 s, 60 °C for 30 s and 72 °C for 60 s. Each reaction was performed at least in triplicate. The specificity of the amplified products was confirmed by a melting peak analysis. Relative mRNA expression for each gene was analyzed using the ΔΔCt method according to Livak [51]. The results were represented as a histogram via GraphPad Prism 5.0 software (San Diego, CA, USA). Sequence primers were reported in Table 2.

### 4.13. Statistical Analysis and Graphical Representation

Data were expressed as mean ± SD. The appropriate one-way ANOVA with the Tukey multiple comparison test was used for statistical comparison between groups. Differences were considered statistically significant at *p* < 0.05 with a 95% confidence interval (* *p* < 0.05, ** *p* < 0.01, *** *p* < 0.001). Analyses and representation as histograms were conducted with the GraphPad Prism 5 software (San Diego, CA, USA).

## Figures and Tables

**Table 1:** Characteristics of patients undergoing the elective surgical procedure.

Patient ID	Gender	Age	BMI
P1	Female	55	28.7
P2	Female	38	29.9
P3	Female	43	29.3
P4	Male	51	28.4
P5	Male	44	28.6

**Table 2:** List of sequence primers to perform RT-qPCR used in this study.

Target Gene	Code Primer Sequence	Provider
PTEN	QT00086933	Qiagen
TIMP-1	QT00084168	Qiagen
MMP-9	QT00040040	Qiagen
PFN-1	QT00079520	Qiagen
TGF-β	QT00000728	Qiagen
IL-10	QT00041685	Qiagen
Ccnd1	QT00495285	Qiagen
Cdkn1B	QT00998445	Qiagen
ki67	QT00014203	Qiagen

## Data Availability

The original contributions presented in this study are included in the article and Supplementary Materials. Further inquiries can be directed at the corresponding authors.

## Supplementary Materials

The following supporting information can be downloaded at: https://www.mdpi.com/article/10.3390/gels11020119/s1. Figure S1: Adipose Mesenchymal cell characterization.

## Abbreviations

The following abbreviations are used in this manuscript:

DFUs	diabetic foot ulcers
sEVs	small extracellular vesicles
ASCs	adipose mesenchymal stem cells
PTEN	phosphatase tensin homolog
PFN-1	profilin-1
VEGF	vascular epidermal growth factor
TIMP	metallopeptidase Inhibitor
MMP-9	metalloproteinase-9
TGF-β	tumor necrosis factor
IL-10	interleukin-10.
ECM	extracellular matrix
GG	gellan gum
EDA	Ethylenediamine
AT	adipose tissue
TTF	tangential flow filtration
DLS	dynamic light scattering
CFSE	5-(and-6)-Carboxyfluorescein Diacetate Succinimidyl ester
HNDF	primary dermal fibroblast, normal, human, neonatal
