# Supplementary figures and images for: Amine-Functionalized Gellan Gum-Based Hydrogel Loaded with Adipose Stem Cell-Derived Small Extracellular Vesicles: An In Vitro Proof of Concept for Enhancing Diabetic Foot Ulcer Healing
Source: Gels. 2025 Feb 6;11(2):119. doi: 10.3390/gels11020119 (PMC11854167; doi:10.3390/gels11020119)

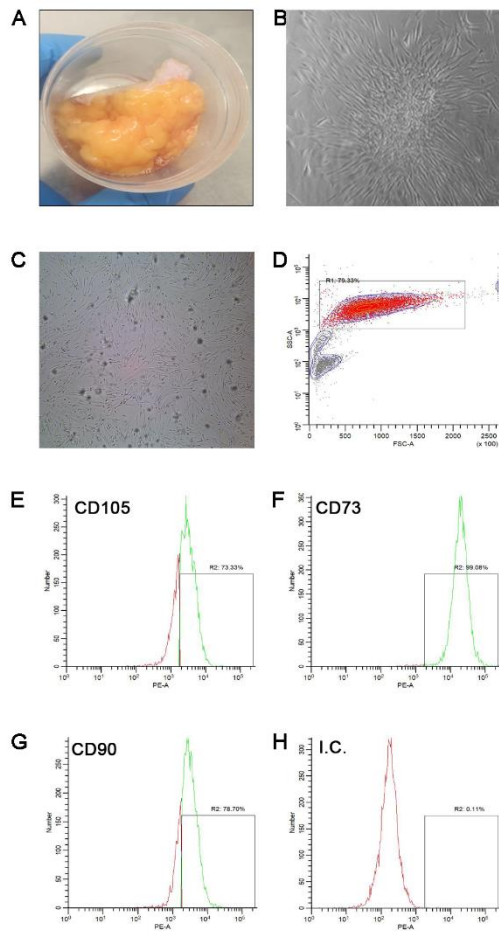

Figure S1: Adipose Mesenchymal cell characterization.

Supplement: Supplementary file 1 [file gels-11-00119-s001.zip › gels-3430864-supplementary.pdf]
